# The Natural Polyphenol Epigallocatechin Gallate Protects Intervertebral Disc Cells from Oxidative Stress

**DOI:** 10.1155/2016/7031397

**Published:** 2016-03-28

**Authors:** Olga Krupkova, Junichi Handa, Marian Hlavna, Juergen Klasen, Caroline Ospelt, Stephen John Ferguson, Karin Wuertz-Kozak

**Affiliations:** ^1^Department of Health Sciences and Technology, Institute for Biomechanics, ETH Zurich, Hoenggerbergring 64, 8093 Zurich, Switzerland; ^2^Department of Orthopaedic Surgery, Fukushima Medical University School of Medicine, Hikarigaoka, Fukushima City, Fukushima 960-1295, Japan; ^3^Prodorso, Walchestrasse 15, 8006 Zurich, Switzerland; ^4^Department of Rheumatology, University Hospital, Zurich, Switzerland

## Abstract

Oxidative stress-related phenotypic changes and a decline in the number of viable cells are crucial contributors to intervertebral disc degeneration. The polyphenol epigallocatechin 3-gallate (EGCG) can interfere with painful disc degeneration by reducing inflammation, catabolism, and pain. In this study, we hypothesized that EGCG furthermore protects against senescence and/or cell death, induced by oxidative stress. Sublethal and lethal oxidative stress were induced in primary human intervertebral disc cells with H_2_O_2_ (total *n* = 36). Under sublethal conditions, the effects of EGCG on p53-p21 activation, proliferative capacity, and accumulation of senescence-associated *β*-galactosidase were tested. Further, the effects of EGCG on mitochondria depolarization and cell viability were analyzed in lethal oxidative stress. The inhibitor LY249002 was applied to investigate the PI3K/Akt pathway. EGCG inhibited accumulation of senescence-associated *β*-galactosidase but did not affect the loss of proliferative capacity, suggesting that EGCG did not fully neutralize exogenous radicals. Furthermore, EGCG increased the survival of IVD cells in lethal oxidative stress via activation of prosurvival PI3K/Akt and protection of mitochondria. We demonstrated that EGCG not only inhibits inflammation but also can enhance the survival of disc cells in oxidative stress, which makes it a suitable candidate for the development of novel therapies targeting disc degeneration.

## 1. Introduction

Degenerative disc disease, characterized by spinal microinstability and lower back pain, is a result of multiple events like age-related degradation of extracellular matrix (ECM), inflammation, or trauma [1, 2]. During degeneration, the intervertebral disc (IVD) undergoes morphological and functional changes such as calcification of endplates, disruption of annulus fibrosus (AF), and loss of water in the nucleus pulposus (NP), which not only impairs disc function but also decreases nutrient supply and causes accumulation of cellular waste products [3–5]. In such situation, cells can compensate for impaired homeostasis, for example, by activation of neovascularization factors, to enhance the availability of nutrients. However, a catabolic environment and abnormal blood supply in the originally avascular tissue can increase reactive oxygen species (ROS) production and oxidative stress [6–10].

ROS are highly reactive molecules with unpaired electrons formed externally or in mitochondria as a normal part of the aerobic metabolism. To ensure a properly maintained redox balance, organisms require a complex, coordinated network of antioxidants from various sources that control ROS generation [11]. Cellular responses to ROS depend on their concentration and duration of exposure as well as the cell type. As signaling molecules, ROS modulate a variety of physiological events, including proliferation, differentiation, host defense, and wound healing [12]. On the other hand, extensive ROS exposure and/or insufficient cellular antioxidant capacity cause deleterious damage, senescence, and cell death [13].

Cellular senescence is described as an irreversible stress-induced cell cycle arrest [14]. Senescence followed by tissue remodeling is beneficial in healthy tissue, where it contributes to the elimination of damaged cells. Furthermore, it is a major protective mechanism against carcinogenesis. However, at the same time, persistent senescence impairs tissue function and leads to inflammation and premature degeneration [15]. Internal DNA damage (telomere shortening) as well as multiple stress signals, including ROS, aberrant oncogene activation, and chemotherapeutic drugs, are able to induce premature senescence via activation of the p53-p21 and/or p16 Ink4a pathway [16]. Although cell and tissue aging has been extensively investigated, the precise mechanisms underlying the development of a senescence phenotype are still unknown [14].

Extensive oxidative stress disrupts the integrity of mitochondria, which can further elicit cell death through three major mechanisms. During apoptosis (1), cytochrome c is released into the cytosol where it activates caspase-9, which allows executioner caspases 3, 6, and 7 to cleave their substrates [17]. Caspase-independent cell death mechanisms, such as regulated necrosis (2) or autophagy (3), are equally important in various pathologies, including IVD degeneration [18, 19]. Similar to senescence, the biological purpose of apoptosis is to eliminate damaged or suboptimal cells [15], although both processes are regulated differently [14]. During apoptosis, a variety of triggers can quickly converge to the executioner effectors through a common mechanism. In contrast, senescence is typically a delayed progressive process, during which various effector mechanisms can each contribute and collectively define the phenotype [14].

The functionality of human IVDs as spinal shock-absorbers gradually decreases throughout lifetime. Senescence of IVD cells, which positively correlates not only with age, but also with the degree of disc degeneration, can be linked to oxidative stress [10, 20–27]. Oxidative stress-induced senescence and death of IVD cells contribute to the local tissue inflammation and the expression of matrix degrading enzymes, hence accelerating loss of proteoglycans [28]. Extensive production of mitochondrial ROS during IVD cell death may further impair disc tissue homeostasis [6, 19, 29–31]. Apart from oxidative stress, also other factors, like nonphysiological mechanical loading, an acidic environment, or autoimmune reactions, can alter the cellular phenotype and enhance premature senescence and cell death in the IVD [25, 32, 33].

Despite the fact that IVD degeneration and back pain represent an economic burden, only symptomatic therapies, like analgesics or invasive surgeries, are currently available. Such therapies do not target the biological causes of disc degeneration and either offer only temporary relief or entail risks and complications. The focus of the current research is to restore the disc function via prevention of premature aging, inhibition of cell death, and enhancement of ECM maintenance with an ultimate goal to prevent low back pain [4, 34]. One approach to compensate the loss of functional disc cells is to apply stem cells embedded in diverse biomaterials mimicking disc tissue, which may support the old disc population and “rejuvenate” the aging disc. However, the implanted cells can in some cases undergo incorrect differentiation, can induce inflammatory reactions, and may not survive due to a prior catabolic environment [35, 36]. Another approach is to enhance survival of the existing intrinsic disc cells using various pharmacological agents such as growth factors or inhibitors of inflammatory pathways [4, 37].

We have previously shown that epigallocatechin 3-gallate (EGCG), a polyphenol naturally occurring in green tea, inhibits inflammatory responses in IVD cells* in vitro* and exhibits analgesic activity against disc-related radiculopathy in experimental animals [38]. The aim of this* in vitro* study was to (1) further investigate whether EGCG exhibits protective effects in mild and high oxidative stress and to (2) investigate the molecular mechanism involved. 

## 2. Methods

### 2.1. DPPH Radical Scavenging Activity Assay

Antioxidant activity of EGCG was determined by measuring its scavenging capacity of the 2,2′-diphenyl-1-picrylhydrazyl (DPPH) radical as previously described [39]. 100 *μ*L of 10–300 *μ*M EGCG was incubated with 500 *μ*L of DPPH (250 *μ*M in ethanol; D9132 Sigma, Buchs, Switzerland) for 1 hour at room temperature (*n* = 3). DPPH radical scavenging activity, which manifests itself as a decrease in absorbance, was measured at 517 nm with a spectrophotometer (Infinite M200 PRO, TECAN Group AG, Männedorf, Switzerland). L-Ascorbic acid (A4403, Sigma) and ethanol (02860, Sigma) in equal amounts were used as positive and negative control, respectively. DPPH radical scavenging activity (%) was calculated as [negative control optical density (OD) − sample OD] *∗* 100/negative control OD. 

### 2.2. Cell Isolation and Cell Culture

The study was approved by the cantonal ethic committee (Kantonale Ethikkommission Zürich EK-16/2005). After informed consent was granted, human NP tissue (grades III–V) was removed from donors undergoing spinal surgeries for degenerative disc disease or disc herniation (*n* = 36). Details about the donors for this study are listed in Table 1. The tissue was enzymatically digested using a mixture of 0.2% collagenase NB4 (17454, Serva, Heidelberg, Germany) and 0.3% dispase II (04942078001, Roche, Basel, Switzerland) for 4–8 hours at 37°C and isolated primary cells were seeded in Dulbecco's Modified Eagle's Medium (DMEM/F12, D8437, Sigma, St. Louis, MO, USA), supplemented with 10% fetal calf serum (FCS, F7524, Sigma), penicillin (50 units/mL), streptomycin (50 *μ*g/mL), and ampicillin (125 ng/mL, 15240-062, Gibco, Carlsbad, CA, USA) and subcultured up to passage 3 using 1.5% trypsin (15090-046, Gibco). Adherent cells in passages 1–3 were used for experiments. Due to the small biopsy size, low proliferation rate, and dedifferentiation of primary IVD cells in monolayer, a single donor cannot be used for all required experiments. Therefore, different donors were randomly assigned to the specific experiments. Cells were seeded on 6-well plates at a density of 1 × 10^5^ cells per well for all senescence experiments. Cells were seeded on 12-well plates at a density of 1 × 10^5^ cells per well for all lethal oxidative stress experiments, except JC-1 staining assay, which was performed on 6-well plates. After seeding, cells were left to adhere overnight.

### 2.3. Experimental Design and Treatments

Oxidative stress was induced with H_2_O_2_ (V800211, Sigma), which initiates the generation of ROS that are able to attack all types of biomolecules [40, 41]. The active concentration of H_2_O_2_ was selected based on the results of a preliminary H_2_O_2_ sensitivity study (*n* = 5). Increasing concentrations of H_2_O_2_ (10–200 *μ*M) were applied for 24 hours and cellular responses were tested by MTT assay. The lethal concentration of H_2_O_2_ (100 *μ*M and 200 *μ*M for 24 hours) as well as the sublethal concentration of H_2_O_2_ which can induce senescent changes without significant cell death (50 *μ*M H_2_O_2_ applied for 2 hours, followed by recovery period) were identified. FCS-free media were used during all oxidative stress treatments to rule out the possible interaction of radicals with FCS components. Previously reported nontoxic EGCG concentrations (5 *μ*M and 10 *μ*M) were applied in this study [38]. 

The effect of EGCG (E4143, Sigma) on premature senescence was tested in three different experimental setups to identify mechanism of EGCG's action (Table 2): (1) to test its antioxidant activity, 10 *μ*M EGCG was applied in the stress phase simultaneously with H_2_O_2_, (2) to test its effects on poststress processes, 10 *μ*M EGCG was added only in the recovery phase, and (3) to combine its potential benefits in both phases, 5 *μ*M EGCG was added in the stress phase and later again in the recovery phase. 

The effect of 10 *μ*M EGCG on cell death was tested directly in the stress phase, without recovery period (Table 2). To further test the involved mechanism, LY294002, an inhibitor of the PI3K/protein kinase B (Akt) pathway (LY, L9908, Sigma), was applied at a concentration of 10 *μ*M, along with EGCG (*n* = 10). PI3K/Akt activator insulin (I9278, Sigma) was used at a concentration of 0.5 *μ*L/mL to confirm functionality and specificity of LY in our experiments (*n* = 10). 

### 2.4. *In Vitro* Model System of Premature Senescence

Premature senescence was induced with sublethal H_2_O_2_. After seeding, cells were starved in FCS-free medium for 2 hours before applying 50 *μ*M H_2_O_2_ for another 2 hours. As a next step, media were replaced by complete media (+10% FCS) to let the cells recover from oxidative stress. Premature senescence was monitored for 15 days with media exchange on days 5 and 10. To verify senescence-associated changes, various tests were performed: senescence-associated *β*-galactosidase activity was tested on days 1, 5, 10, and 15 after stress (*n* = 5). The number of viable cells was determined by Trypan blue exclusion test on days 8 and 15 (*n* = 5). Cellular metabolic activity was measured by MTT assay and expression/activity of senescence-associated proteins p21 and p53 was analyzed by immunoblotting on day 15 (*n* = 5). In a separate experiment on day 8, trypsin-detached cells were reseeded again to check their ability to adhere, which reflects general cellular fitness. 

### 2.5. Senescence-Associated SA *β*-galactosidase Assay

Senes-cence-associated *β*-galactosidase (SA *β*-gal), which accumulates in aging cells, serves as a reliable marker of senescence* in vitro *[42]. Senescence was induced with 50 *μ*M H_2_O_2_, followed by EGCG treatments as described above (*n* = 5 for each experimental setup). For SA *β*-gal analysis, staining solution containing 40 mM Na_3_ citrate × 2H_2_O + 5 mM K_4_Fe(CN)_6_ + 5 mM K_3_Fe(CN)_6_ + 0.15 M NaCl + 2 mM MgCl_2_ in H_2_O was prepared and its pH was adjusted to 6 by adding 0.5 M NaH_2_PO_4_ (all from Sigma). X-gal (11680293, Roche) was dissolved in dimethylformamide (33120, Sigma) to prepare a 20 mg/mL stock solution. Adherent cells were washed twice with PBS and fixed with 3% paraformaldehyde (30525894, Acros, Geel, Belgium) in PBS for 5 minutes at room temperature. After the fixation, cells were washed twice with PBS and a mixture of X-gal + staining solution in a 1 : 20 ratio was added. After 18 hours, the staining solution was removed and cells were washed two times in distilled water and passed through graded ethanol (75%, 95%, 99%), 1 minute each. After air-drying, micrographs were taken from 3 random spots of each well using light microscopy. The number of all cells (minimum 300) and SA *β*-gal positive cells was counted by processing images with GIMP (http://www.gimp.org/) and ImageJ (http://imagej.nih.gov/ij/). The percentage of SA *β*-gal positive cells in each treatment group was calculated as the number of SA *β*-gal positive cells/number of all cells *∗* 100. SA *β*-gal positive cells in the treatment groups were displayed relative to the control groups, due to the high interdonor variability in basal SA *β*-gal staining.

### 2.6. Trypan Blue Exclusion Test

Trypan blue staining, which distinguishes viable and dying cells, was performed to analyze the total number of viable cells at the end of each experiment. Senescence was induced with 50 *μ*M H_2_O_2_ and EGCG treatments were performed as described above (*n* = 5 for each experimental setup). Cells were harvested using 1.5% trypsin into the complete media, an aliquot was mixed 1 : 1 with 0.4% Trypan blue dye (93595, Fluka), and the cell suspension was immediately analyzed on the grids of the hemacytometer (DHC-N01, Thermo Scientific). The absolute number of nonstained (viable) cells was determined in each group to make a comparison with the total number of seeded cells (1 × 10^5^ cells per well). Nonviable cells, which took up Trypan blue dye, were excluded from the analysis. 

### 2.7. Metabolic Activity Measurement

Metabolic activity, which reflects cellular viability, was determined using the MTT assay. Following seeding, sublethal or lethal oxidative stress was applied and the treatments were performed as described above (*n* = 5 for sublethal oxidative stress experiments, *n* = 10 for lethal oxidative stress experiment, and *n* = 10 for inhibition experiments). After 24 hours or 10 days, fresh MTT (3-[4,5-dimethylthiazol-2-yl]-2,5-diphenyl tetrazolium bromide, M5655, Sigma) solution in PBS (0.5 mg/mL) was added and kept for 3 hours at 37°C. MTT was discarded, cells were lysed in DMSO (D8418, Sigma), and the absorbance was measured at 565 nm. Metabolic activity was calculated relative to the untreated control (100%).

### 2.8. Immunoblotting

Treatments were performed as described above. Cells were harvested after 15 min treatment for lethal oxidative stress experiments (*n* = 5) or after 10 days and 15 days for sublethal oxidative stress experiments (*n* = 5). Whole cell lysates were prepared in RIPA buffer (89900, Thermo Scientific, Waltham, MA, USA) according to the producer's instructions, mixed with Laemmli buffer (S3401, Sigma), heated (99°C, 5 minutes), and then loaded onto 12% SDS polyacrylamide gels. Separated proteins were transferred onto polyvinylidene difluoride (PVDF) membranes (RPN303F, GE Healthcare, Little Chalfont, UK) and membranes were blocked in 5% nonfat milk in Tris-buffered saline-Tween (TBS-T) for 1 hour at room temperature. Primary antibodies were applied overnight at 4°C. After washing in 1% nonfat milk in TBS-T (3 × 10 min), membranes were incubated with a secondary antibody conjugated to horseradish peroxidase (HRP) for 1 hour at room temperature and washed in 1% nonfat milk in TBS-T (3 × 10 min). Visualization was performed on medical X-ray film (28906836, GE Healthcare), using a chemiluminescence kit West Dura (34076, Thermo Scientific). Tubulin was used as a loading control. Antibodies and dilutions were as follows: phospho-p53 (Ser15) 1 : 1000 (9284, Cell Signaling, Danvers, MA, USA); p21 1 : 1000 (2947, Cell Signaling); phospho-Akt (Ser473) 1 : 1000 (9271, Cell Signaling); Akt 1 : 1000 (9272, Cell Signaling); p16 Ink4a (p16) 1 : 1000 (ab108349, Abcam); *α*-Tubulin 1 : 1000 (2144, Cell Signaling); and mouse anti-rabbit IgG HRP 1 : 5000 (7074, Cell Signaling).

### 2.9. Analysis of Mitochondrial Transmembrane Potential (JC-1 Staining)

JC-1 fluorochrome (5,5′,6,6′-tetrachloro-1,1′,3,3′-tetraethylbenzimidazolcarbocyanine iodide) allows for monitoring changes in mitochondrial transmembrane potential, which precedes cell death. In physiological conditions, JC-1 accumulates in the intact mitochondrial membrane, where it forms pronounced red aggregates. Once mitochondrial membrane potential is disturbed, the red fluorescence decreases, which is accompanied by an increase in green fluorescence of the free JC-1 form. A substantial increase in the green fluorescence  ( = reduced red/green ratio) suggests a breakdown of mitochondrial potential and activation of cell death.

Lethal oxidative stress was applied and EGCG treatment was performed as described above (*n* = 10). After 24 hours, 1 *μ*g/mL of JC-1 (T0046, Chemodex, St. Gallen, Switzerland) diluted in FCS-free media was added to the cells and kept at 37°C in the dark for 30 minutes. Then, cells were washed (3 × PBS) and images of 3 random spots per well were taken immediately with a fluorescence microscope (Olympus IX51, Volketswil, Switzerland). The ratio of red/green fluorescence was calculated using ImageJ analysis (http://imagej.nih.gov/ij/).

### 2.10. Propidium Iodide/Annexin V-FITC Staining

The membrane of living cells is impermeable for Propidium Iodide (PI), a red fluorescent dye which intercalates into DNA. Annexin V-FITC (A) binds membrane phosphatidylserine, which is exposed to the extracellular environment as one of the early apoptotic events. Flow cytometry analysis of PI/A staining allows distinguishing viable cells (PI-negative/A-negative), cells in early apoptosis (PI-negative/A-positive), and dead cells (PI-positive/A-positive) [43].

Lethal oxidative stress was applied and EGCG treatment was performed as described above (*n* = 5). After 24 hours, supernatants were collected into prechilled tubes and placed on ice. Cells were washed with PBS, detached from cell culture wells with 1.5% trypsin, and transferred into the corresponding prechilled tubes with matching supernatants. All the further steps were performed on ice. Cell-supernatant mixtures were centrifuged (250 ×g/5 min, 4°C), supernatants were discarded, and 400 *μ*L of Annexin Binding Buffer (556454, BD Biosciences, Franklin Lakes, NJ, US) was added to the pellets and mixed. After the addition of 1 *μ*L of PI (556463, BD Biosciences) and 2.5 *μ*L of Annexin (556420, BD Biosciences), tubes were kept in the dark for 15 minutes. Flow cytometry was performed immediately using the cytometer FACS ARIA III (BD Biosciences). Results were analyzed with FlowJO (http://www.flowjo.com/) and are displayed as percentage of viable cells (PI^−^/A^−^) in each treatment group. Unstained cells, cells stained with Annexin V-FITC alone, and cells stained with PI alone were used as controls to set up quadrants.

### 2.11. Gene Expression Analysis (RT-qPCR)

The effect of sublethal oxidative stress on the activation of senescence-associated secretory phenotype (SASP) was tested by means of gene expression of interleukin-6 (IL-6), interleukin-8 (IL-8), matrix metalloproteinase 1 (MMP1), matrix metalloproteinase 3 (MMP3), and matrix metalloproteinase 13 (MMP13). These genes are typically expressed in a proinflammatory and catabolic environment during inflammation-related disc degeneration [44, 45]. IVD cells were seeded on 6-well plates and senescence was induced as described above (*n* = 4). After 24 hours, RNA was extracted with the Trizol/chloroform method according to the manufacturer's instructions (15596-018, Invitrogen, Carlsbad, CA, USA) and 1 *μ*g was reverse transcribed to cDNA using a reverse transcription kit (4374966, Applied Biosystems). cDNA was then mixed with primers and master mix (4352042, Applied Biosystems) and gene expression was measured using real-time PCR. The following primers were used: TATA box binding protein (TBP) Hs00427620_m1, IL-6 Hs00174131_m1, IL-8 Hs00174103_m1, MMP1 Hs00233958_m1, MMP3 Hs00968308_m1, and MMP13 Hs00233992_m1. Data was analyzed with the comparative Cq method (2^−ΔΔCq^, housekeeping gene TBP). Results are presented as a fold change relative to the untreated control group.

### 2.12. Statistical Analysis

A power calculation (80% power) determined the required sample size (*n* = 5). Data normality was tested by Schapiro-Wilk test. Statistical significance between two groups was analyzed using Student's *t*-test. Statistical significance between more than two groups was evaluated by ANOVA with Tukey* post hoc* test. The number of donors used for each experiment is listed in Methods. Mean and SEM are displayed in graphs and asterisks represent a significance level of *p* < 0.05.

## 3. Results

### 3.1. Validation of the* In Vitro* Model System of Premature Senescence of IVD Cells

**Figure 1:**
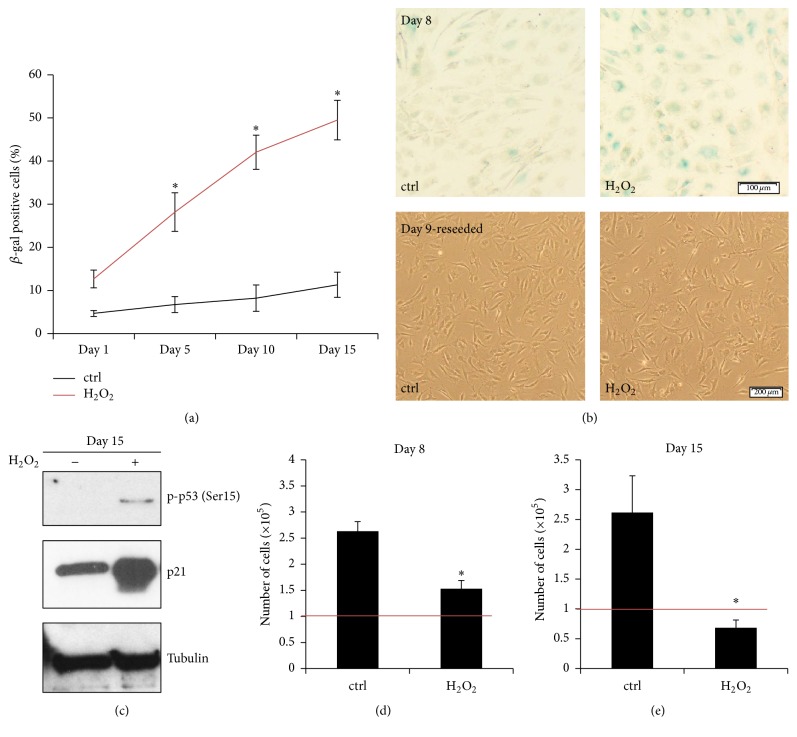
*In vitro* model system of stress-induced premature senescence. Sublethal oxidative stress (50 *μ*M H_2_O_2_) with subsequent recovery period activated premature senescence of IVD cells* in vitro*. (a) Percentage of SA *β*-gal-positive cells in the H_2_O_2_ treatment group gradually increased during 15 days after stress (*n* = 5). (b) Upper part: representative images of SA *β*-gal staining of the untreated (ctrl) and the H_2_O_2_-treated cells on day 8 after stress, showing senescent (blue) cells. (b) Lower part: representative images of reseeded cells on day 9, confirming general cellular fitness. (c) Phosphorylation of p53 (Ser15) and expression of p21 in the H_2_O_2_ treatment group on day 15 after stress indicated cellular senescence. ((d), (e)) Proliferative capacity, displayed as number of cells on days 8 and 15 after stress, was reduced in the H_2_O_2_ groups. On day 15, the number of cells in the H_2_O_2_ treatment group decreased below the seeding number (1 × 10^5^ cells per well, depicted as red line), suggesting ongoing cell death. Asterisks indicate statistical significance at *p* < 0.05 (ANOVA, Tukey* post hoc*, and Student's *t*-test).

Based on the sensitivity study, 50 *μ*M H_2_O_2_ applied for 2 hours and followed by a recovery period up to 15 days was chosen to induce premature senescence of IVD cells without significant cell death (Supplementary Figure  1 in Supplementary Material available online at http://dx.doi.org/10.1155/2016/7031397). Analysis of senescence-associated changes was performed on days 1, 5, 8, 10, and 15 after stress. In the H_2_O_2_ treatment group, the number of SA *β*-gal positive cells increased significantly from day 1 (12.67 ± 2.05%) up to day 15 (49.53 ± 4.58%) after stress, compared with the untreated controls (Figure 1(a)). Representative images of SA *β*-gal staining on day 8 and reseeded cells on day 9 are shown (Figure 1(b)). Reseeded cells in the H_2_O_2_ treatment group were able to adhere, which confirmed a good general cellular fitness; however, altered cellular morphology suggested an aging cell population.

Phosphorylation of the stress-induced protein p53 (Ser15) and enhanced expression of the cell cycle inhibitor p21 on day 15 in the H_2_O_2_ treatment group indicated oxidative damage (Figure 1(c)). In order to compare the replicative potential of stressed and control cells, the living cells were counted on days 8 and 15 by Trypan blue exclusion test. The cell number on day 8 in the H_2_O_2_ group (1.53 ± 0.16 × 10^5^ cells/well) was significantly lower than in the control group (2.63 ± 0.18 × 10^5^ cells/well), confirming oxidative stress-induced loss of proliferative capacity. Cell death in the H_2_O_2_ group on day 8 did not occur, as the number of cells was higher than the cell seeding density on day 0 (1 × 10^5^ cells/well, marked as red line) (Figure 1(d)). However, on day 15, the number of cells in the H_2_O_2_ treatment group decreased below the seeding density (0.68 ± 0.13 × 10^5^ cells/well versus 1 × 10^5^ cells/well, marked as red line), indicating that the cells slowly started to die from the ROS exposure (Figure 1(e)). As cell death may hamper data interpretation, all subsequent experiments were performed for a maximum of 10 days.

The expression of inflammatory genes in the H_2_O_2_ treatment group, measured on day 1, was not significantly elevated (Supplementary Figure  1).

### 3.2. EGCG Inhibited Senescence-Associated *β*-galactosidase Accumulation in Sublethal Oxidative Stress

**Figure 2:**
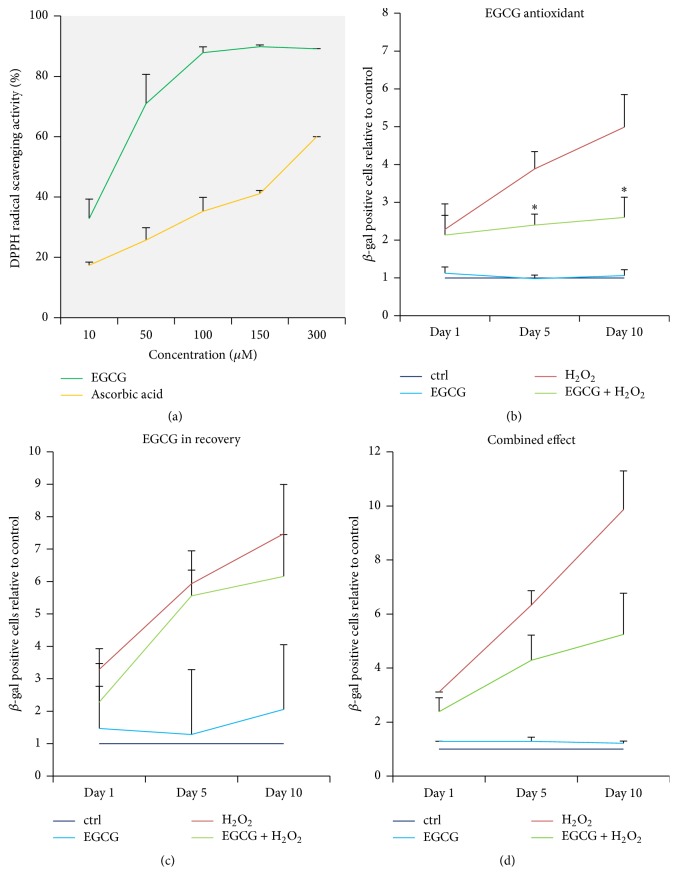
As an antioxidant, EGCG inhibited senescence-associated *β*-galactosidase accumulation. (a) EGCG exhibited increasing 2,2-diphenyl-1-picrylhydrazyl (DPPH) radical scavenging activity between 10 and 100 *μ*M, which confirmed its antioxidant properties. Ascorbic acid in the same concentration was used as positive control (*n* = 3). ((b)–(d)) Oxidative stress was induced with 50 *μ*M H_2_O_2_ for 2 hours and cellular senescence was measured during the following 10 days. 10 *μ*M EGCG inhibited SA *β*-gal accumulation when added to the oxidative stress phase, when its antioxidant activity was confirmed (*n* = 5). (c) 10 *μ*M EGCG added to the recovery phase did not influence SA *β*-gal accumulation compared to the H_2_O_2_-only group (*n* = 5). (d) EGCG combined in both phases (5 + 5 *μ*M) did not significantly inhibit SA *β*-gal accumulation, although a trend is visible (*n* = 5). Asterisks indicate statistical significance at *p* < 0.05 (ANOVA, Tukey* post hoc*).

Previously reported antioxidant properties of EGCG were confirmed using 2,2-diphenyl-1-picrylhydrazyl (DPPH) radical assay. EGCG exhibited a dose-dependent radical scavenging activity between 10 and 100 *μ*M. As a common antioxidant, ascorbic acid at the same concentrations was used as a positive control (Figure 2(a)) [46]. Apart from its antioxidant effects, EGCG may also possess other beneficial functions. Therefore, the effect of EGCG on the accumulation of SA *β*-gal was tested in 3 different experimental setups displayed in Table 2, namely, (1) antioxidant activity by adding EGCG in the stress phase, (2) activity on poststress signaling by adding EGCG in the recovery phase, and (3) potential combined effect by adding EGCG in both phases.

The percentage of SA *β*-gal positive cells was evaluated on days 1, 5, and 10 after stress. EGCG significantly inhibited the accumulation of SA *β*-gal on days 5 and 10 when added during the stress phase, indicating that an antioxidant mechanism is involved (Figure 2(b)). EGCG did not influence the rate of SA *β*-gal accumulation during the recovery phase, suggesting that the interaction of EGCG with poststress signaling is less relevant (Figure 2(c)). The presence of EGCG in both phases did not provide significant protection against oxidative stress, which is most likely caused by insufficient ROS neutralization, as lower concentrations of EGCG (5 *μ*M) were used in the stress phase of this setup. However, an apparent trend towards statistical significance was detectable (Figure 2(d)).

### 3.3. EGCG Did Not Protect IVD Cells from Loss of Proliferative Capacity in Sublethal Oxidative Stress

**Figure 3:**
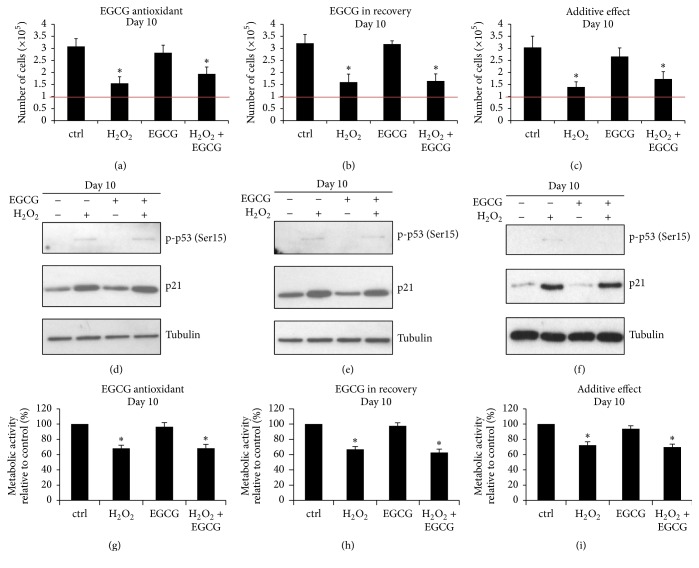
EGCG did not influence reactive oxygen species-induced loss of proliferative capacity. Senescence of IVD cells was induced with 50 *μ*M H_2_O_2_ for 2 hours and proliferative capacity was evaluated on day 10 after stress. ((a)–(c)) Over a period of 10 days, the H_2_O_2_-treated cells proliferated significantly less than the cells in control groups. The number of cells in the H_2_O_2_ and H_2_O_2_ + 10 *μ*M EGCG treatment groups did not significantly differ in either experimental setup (*n* = 5). ((d)–(f)) The activity of p53 and the expression of p21 were not significantly affected by EGCG in either experimental setup (*n* = 5). ((g)–(i)) Metabolic activity in the H_2_O_2_ and the H_2_O_2_ + EGCG treatment groups did not significantly differ, indicating reduced proliferative capacity in all stress groups (*n* = 5). Asterisks indicate statistical significance at *p* < 0.05 versus control group (ANOVA, Tukey* post hoc*).

The effects of EGCG on proliferation and expression of the senescence markers p53 and p21 were also tested in each experimental setup: (1) EGCG as an antioxidant, (2) EGCG in recovery, and (3) combined effects. In order to compare the proliferative capacity of H_2_O_2_-treated and H_2_O_2_ + EGCG-treated cell populations, the number of viable cells on day 10 of recovery period was determined in each treatment group. Untreated and EGCG-treated cells proliferated, but all stress groups showed significantly lower cell numbers, regardless of the presence of EGCG (Figures 3(a)–3(c)). On day 10 after stress, p53 was slightly phosphorylated in all stress groups. Expression of p21 was induced by H_2_O_2_ but also not affected by the presence of EGCG, hence indicating that exogenous ROS were not entirely neutralized by 10 *μ*M EGCG (Figures 3(d)–3(f)).

As both nonsenescent and senescent cells are metabolically active, detected metabolic activity on day 10 corresponds to the number of cells in each treatment group: stress groups showed significantly lower metabolic activity than untreated controls. No significant difference between the H_2_O_2_ and H_2_O_2_ + EGCG treatment groups was detected in either experimental setup, confirming that EGCG did not protect IVD cells from loss of proliferative capacity (Figures 3(g)–3(i)). Metabolic activity of cells on day 10 is displayed relative to the control group (100%).

### 3.4. EGCG Protected IVD Cells from Lethal Oxidative Stress via Inhibition of Mitochondrial Membrane Depolarization

Based on the sensitivity study, 100 *μ*M and 200 *μ*M H_2_O_2_ applied for 24 hours were identified to trigger significant cell death (Supplementary Figure  1). As such ROS doses may induce excessive mitochondria membrane depolarization and unrepairable damage, the general prosurvival effect of EGCG can be tested.

**Figure 4:**
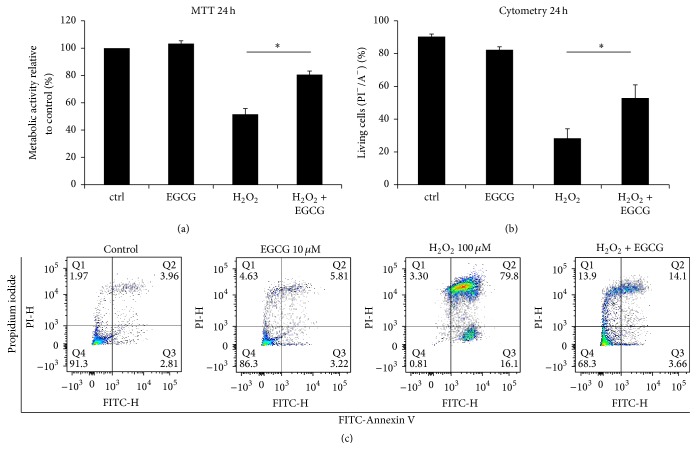
EGCG significantly inhibited reactive oxygen species-induced cell death. Cell death was induced by lethal concentration of H_2_O_2_ (100 *μ*M) applied for 24 hours. (a) 10 *μ*M EGCG significantly reversed the detrimental effects of H_2_O_2_ on metabolic activity, measured by MTT assay (*n* = 10). (b) 10 *μ*M EGCG also significantly increased cell viability in oxidative stress, as measured by Propidium Iodide/Annexin staining (*n* = 5). (c) Visualization of representative Propidium Iodide/Annexin V staining measured by flow cytometry. Asterisks indicate statistical significance at *p* < 0.05 (Student's *t*-test).

Extensive oxidative stress (100 *μ*M H_2_O_2_) significantly decreased the metabolic activity of IVD cells (51.49 ± 5.64%), compared with the control group (100%), while the addition of EGCG reversed this effect (80.58 ± 4.31%) (Figure 4(a)). More specifically, PI/A staining confirmed significantly higher number of living cells in the H_2_O_2_ + EGCG treatment group (52.91 ± 8.02%) compared to the H_2_O_2_-only group (28.31 ± 5.83%) (Figure 4(b)).

**Figure 5:**
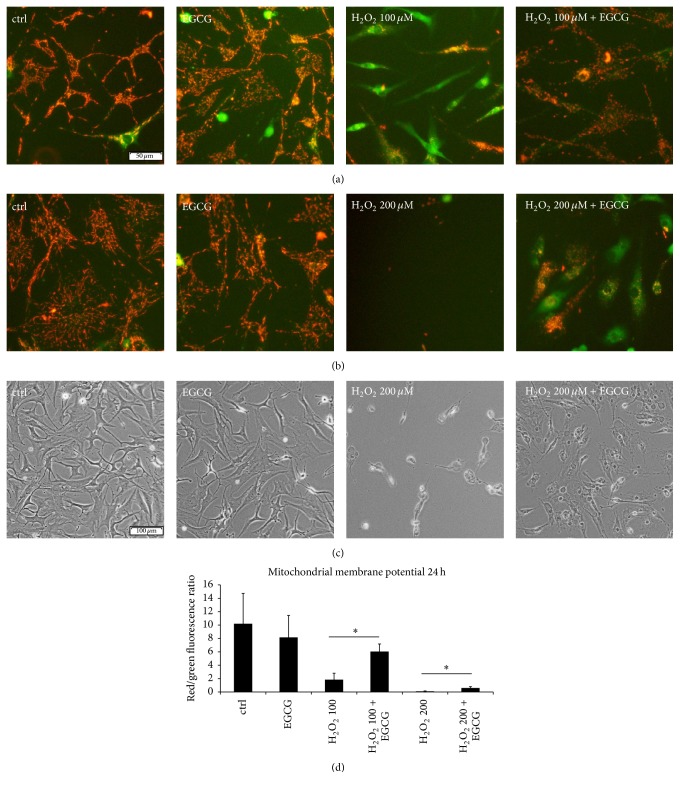
EGCG inhibited the loss of mitochondrial membrane potential. Cell death was activated by lethal concentrations of H_2_O_2_ (100 *μ*M, 200 *μ*M) applied for 24 hours. (a) 10 *μ*M EGCG significantly inhibited mitochondrial membrane depolarization induced with 100 *μ*M H_2_O_2 _(*n* = 10). (b) 10 *μ*M EGCG significantly inhibited mitochondrial membrane depolarization induced with 200 *μ*M H_2_O_2_ (*n* = 10). (c) Cellular morphology of the H_2_O_2_ + EGCG treatment group was different from the control group: signs of membrane blebbing and nuclear condensation indicated that cell death was not completely inhibited (*n* = 5). (d) Ratio of red/green fluorescence showing the loss of mitochondrial membrane potential in the H_2_O_2_ treatment groups. Asterisks indicate statistical significance at *p* < 0.05 (ANOVA, Tukey* post hoc*).

During lethal oxidative stress, mitochondria lose their transmembrane potential, which leads to the release of mitochondrial ROS and serves as a proapoptotic signal. EGCG may possibly interact with ROS on the mitochondrial membrane and prevent further ROS leakage. To test this hypothesis, the JC-1 dye, which forms red aggregates in intact mitochondria, was used. H_2_O_2_ in a concentration of 100 *μ*M (Figure 5(a)) and 200 *μ*M (Figure 5(b)) caused extensive mitochondrial membrane depolarization, while the addition of EGCG prevented this effect. The red/green fluorescence ratio of the untreated (10.19 ± 4.54) and the EGCG-treated (8.15 ± 3.29) cells was significantly higher than the ratio of both H_2_O_2_ treatment groups (100 *μ*M: 1.85 ± 0.96, 200 *μ*M: 0.09 ± 0.07). Addition of EGCG significantly increased the ratio in H_2_O_2_ treatment groups to 6.05 ± 1.14 (100 *μ*M) and 0.63 ± 0.2 (200 *μ*M) (Figure 5(d)). Membrane blebbing and nuclear shrinkage were detected in the H_2_O_2_ + EGCG treatment group, confirming that the cells were not completely protected from deleterious ROS and cell death was possibly only delayed (Figure 5(c)).

### 3.5. EGCG Increased Survival of IVD Cells in Lethal Oxidative Stress via PI3K/Protein Kinase B (Akt) Activation

**Figure 6:**
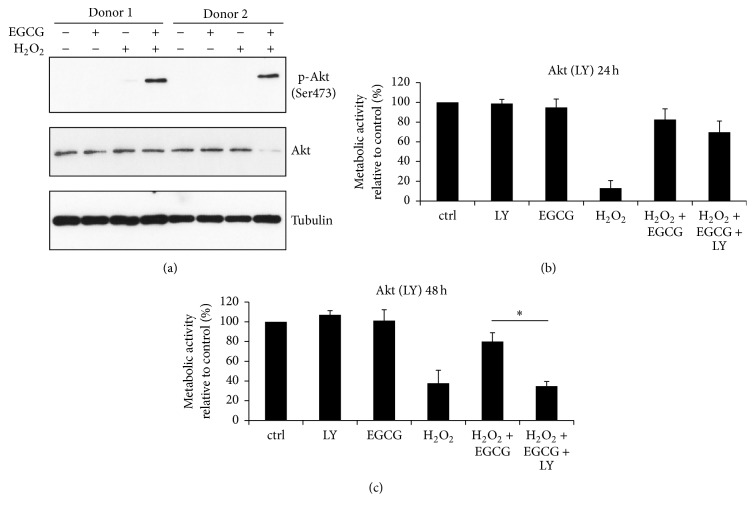
PI3K/Akt is important for the protective function of EGCG under the lethal oxidative stress. Cell death was activated by 200 *μ*M H_2_O_2_. (a) After 15 minutes of cotreatment, 10 *μ*M EGCG activated PI3K/Akt (*n* = 5). (b) After 24 hours, no significant difference in metabolic activity between the H_2_O_2_ + EGCG and the H_2_O_2_ + EGCG + LY groups was detected (*n* = 5). (c) After 48 hours, LY completely abolished the protective effects of EGCG, underlying the importance of the PI3K/Akt pathway in the survival of IVD cells (*n* = 5). Asterisks indicate statistical significance at *p* < 0.05 (ANOVA, Tukey* post hoc*).

To further explore the mechanism underlying the action of EGCG, the activity of the prosurvival PI3K/Akt pathway was studied. As shown by immunoblotting, EGCG activated Akt under oxidative stress conditions (Figure 6(a)). The biological relevance of this finding was tested using the PI3K/Akt-specific inhibitor LY294002 (LY). Depending on the type of stimulus, Akt can activate various downstream signaling pathways inducing proliferation, glucose metabolism, or survival. Akt activator insulin, which stimulates cell proliferation, was used as positive control to confirm the activity and specificity of LY. As expected, insulin modestly increased the proliferation of IVD cells (117.79 ± 2.91% versus 100% in control), whereas the addition of LY abolished this effect (108.16 ± 2.86%), hence confirming the activity of LY for further PI3K/Akt pathway tests (Supplementary Figure  1).

PI3K/Akt inhibition with LY did not significantly alter the prosurvival effect of EGCG at 24 hours, as measured by MTT assay (Figure 6(b)). However, at longer time points (48 hours), the difference in the metabolic activity between the H_2_O_2_ + EGCG (80.01 ± 8.93%) and the H_2_O_2_ + EGCG + LY (34.81 ± 4.69%) treatment group became significant (Figure 6(c)), indicating that EGCG-based activation of the PI3K/Akt pathway plays indeed an important role in the survival of IVD cells under the oxidative stress.

## 4. Discussion

A stress-related decrease in the number of viable cells and phenotypic changes of living cells are two major events in the process of IVD degeneration that warrant therapeutic targeting [32]. Oxidative stress-induced senescence and cell death* in vitro* can represent mechanisms involved in human disc aging* in vivo* [20, 21, 28]. We hypothesized that the natural polyphenol EGCG can inhibit oxidative stress-induced senescence and/or cell death of IVD cells and we investigated which mechanisms may be involved in its action.

*In vitro* as well as* in vivo*, EGCG can directly interact with ROS or operate in an indirect manner by inhibiting ROS generating enzymes or by chelating potentially prooxidant metal ions [47]. Nevertheless, antioxidant activity is not the only biological effect of EGCG. In fact, many of its health-beneficial functions are independent of redox reactions. Depending on the cell type, EGCG can interact with proteins and phospholipids in the plasma membrane, regulate signal transduction and gene expression and DNA methylation, and modulate mitochondrial function and autophagy [48].

Results of the radical scavenging assay confirmed the previously reported antioxidant activity of 10 *μ*M EGCG, which can neutralize 32.89 ± 6.39% of 250 *μ*M DPPH radicals. Although higher concentrations of EGCG exhibited better ROS trapping, they were not used in this study to avoid possible unfavorable interactions with cell culture media components. Dose-dependent oxidation of polyphenols and subsequent ROS generation was reported as a common artifact of* in vitro* cell culture [49, 50]. The thus induced toxicity has no significance* in vivo*, because of the presence of plasma antioxidants, but can interfere with research hypotheses via increasing unspecific stress in cell culture. From this point of view, 10 *μ*M EGCG was considered active, yet experimentally safe.

We developed an* in vitro* model system of premature senescence of IVD cells, induced by sublethal oxidative stress. Due to the vastly heterogeneous nature of the senescence phenotype, the use of various markers is required to precisely identify senescence in a given cell population [14]. The expression of certain markers, such as p21, can also increase during* in vitro* cell culture as an artifact (Supplementary Figure  1). Senescent cells commonly undergo cell cycle arrest, start expressing SA *β*-gal, exhibit a senescence-associated secretory phenotype (SASP), and activate p53-p21 and/or p16-RB pathways [15, 27]. The morphology of senescent cells can differ from their young counterparts: cells become bigger, with prominent nucleoli and numerous cytoplasmic vacuoles [51]. Sublethal doses of the widely used oxidative stress inducer H_2_O_2_ inhibited proliferation and activated the p53-p21 pathway and SA *β*-gal accumulation in IVD cells, proving the suitability of the system to test potential antisenescence compounds.

EGCG did not influence the progress of senescence when applied during the recovery phase, suggesting no interaction with poststress signaling. During recovery after oxidative stress, cells can undergo several changes, one of which can be the activation of gene expression towards the senescence-associated secretory phenotype (SASP). The SASP is mediated, for example, by the transcription factor nuclear factor *κ*B (NF-*κ*B) and many other redox-sensitive transcription factors, which can induce the expression of proinflammatory cytokines, proteases, and catabolic enzymes [11, 15, 37]. We have shown before that EGCG inhibited IL-1*β*-activated inflammatory responses in the IVD [38], implying that EGCG may act via similar mechanisms also in the ROS-activated SASP. However, sublethal oxidative stress in our settings did not increase the expression of inflammatory markers (IL-6, IL-8) and catabolic enzymes (MMP1, MMP3, and MMP13). Therefore, the effect of EGCG on SASP could not be evaluated (Supplementary Figure  1). Previously, stress-induced SASP was activated in IVD cells under different culture conditions [27]; in our setup either longer time point or higher ROS concentration may potentially be required.

As an antioxidant, 10 *μ*M EGCG inhibited accumulation of SA *β*-gal but did not protect IVD cells from loss of proliferative capacity, suggesting that the exogenous radicals were not entirely neutralized. The increase in SA *β*-gal expression/activity results from the need to compensate for the accumulation of damaged macromolecules and organelles in lysosomes. As the expression of SA *β*-gal is not required for senescence, we hypothesize that EGCG in the antioxidant setup can ameliorate the increase in lysosome dysfunction, rather than inhibiting a complex senescence phenotype. In accordance with that, EGCG has been shown to promote macroautophagy and lysosome recycling of accumulated intracellular materials [52, 53]. Nevertheless, the role of EGCG in enhancing cellular waste-disposal mechanisms is still controversial and presumably cell-type specific.

Since EGCG inhibited SA *β*-gal accumulation in the antioxidant setup and not in the recovery setup, one could expect similar responses when combining EGCG in both stress and recovery phase. However, in the third setup, SA *β*-gal assay showed only a trend towards the statistical significance, possibly because a lower EGCG concentration was applied in the stress phase, in order to avoid potential toxic effects mentioned above [54].

As p16 is another important senescence marker, its expression was initially tested in IVD cells treated with EGCG in the antioxidant settings, on day 10 (*n* = 4). Because of the fact that p16 expression in H_2_O_2_ treatment groups was detected only in 1 out of 4 donors, the experiments on p16 expression were not continued (Supplementary Figure  2). In fact, the relative contributions of the p53-p21 and p16-RB pathways to the senescence growth arrest may vary, depending on the type and level of stress [55]. Moreover, in different cell types, the transition of temporal to stable cell cycle arrest can involve either an activation of p21 or p16 or an activation of both [55]. Human IVD cells are generally slowly proliferating and responding cell type in which ROS can possibly induce p16 with delayed kinetics. p16, if activated, subsequently provides a second barrier to stop cell proliferation and plays a role in maintaining senescence, rather than in its initiation, as suggested before [56, 57], which may explain its low expression in our study.

To test whether EGCG can influence the expression of p16, SAOS-2 cells, with constitutive expression of p16, were treated with 10 *μ*M EGCG for 24 hours. This experiment showed no influence of EGCG on the expression of p16 in SAOS-2 (Supplementary Figure  2).

Lethal oxidative stress causes unrepairable damage on mitochondrial membranes, leading to extensive ROS leakage and quick activation of death signals [58] (Supplementary Figure  3). Two independent viability assays revealed that EGCG can significantly increase survival of IVD cells in lethal oxidative stress. As a primary target of oxidative stress, mitochondria quickly lose their membrane integrity. We demonstrated that EGCG can delay the onset of mitochondrial membrane depolarization, hence protecting mitochondria from ROS leakage. Similar antioxidant effects of EGCG were previously described, for example, in the mice liver injury* in vivo* model [59] or in rat retinal primary cells* in vitro* [60]. 

As an exogenous oxidizing agent, H_2_O_2_ produces hydroxyl radicals, which can efficiently react with all cellular components. On the other hand, damaged mitochondria generate mainly superoxide, followed by lower amounts of hydrogen peroxide and hydroxyl radicals [61, 62]. Therefore, EGCG can affect mitochondrial ROS leakage by being a good superoxide anion radical scavenger [62], but it may neutralize other ROS species less efficiently (Supplementary Figure  3). As previously reported, EGCG can also activate superoxide dismutase expression, another mechanism to counteract exclusively superoxide anion radicals and to protect mitochondria [63, 64]. 

Interestingly, EGCG activated PI3K/Akt under lethal oxidative stress, an ability that was important for IVD cell survival. We suggest that the reason why EGCG alone did not activate PI3K/Akt pathway can be an absence of the stress signal (H_2_O_2_). In nonstressed cells, the activity of prosurvival pathways (such as PI3K/Akt) is negatively regulated by their inhibitors (such as PTEN). In stressed cells, the activity of intracellular kinase inhibitors can be reduced [65] in order to facilitate activation of the prosurvival kinases. However, the prosurvival kinases can fully function only upon external activation [66]. Interestingly, it has been shown that EGCG interacts with various membrane receptors [48, 67]. We therefore hypothesize that EGCG-mediated interaction upstream of Akt, in combination with stress-mediated reduction in the activity of intracellular kinase inhibitors, can lead to complete PI3K/Akt activation and prosurvival effects in H_2_O_2_-treated cells. 

Previously, Akt gene expression has been correlated with increased disc cell proliferation* in vitro* [68] and lumbar disc herniation* in vivo* [69]. More importantly, active PI3K/Akt signaling enhanced prosurvival capacity of disc cells* in vitro* [70] and antagonized disc degeneration* in vivo* [71]. The PI3K/Akt pathway can therefore represent another attractive therapeutic goal in disc degeneration, which can be targeted by EGCG. 

In this study, 2D cell culture was employed to ensure applicability of cell analysis assays. Adherent* in vitro* system, in contrast to 3D, reduced cell loss during analyses, which is particularly important for stress experiments. The 2D system also allowed quick and precise staining of organelles in living cells. While results are very clear, it remains open whether cells react similarly in their natural 3D environment. This study is furthermore limited by the interdonor variability of disc tissue biopsies, originating from differences in age and degeneration grades [72]. Nevertheless, the use of primary cells during first three passages represents a system more close to human tissue than a stabilized cell line. The interdonor variability issue can be corrected by displaying results relative to a control group, instead of using absolute numbers. The main third limitation is that the effect of EGCG during the recovery phase of senescence was tested in complete media, which could possibly influence the activity of EGCG.

## 5. Conclusions

This study showed that EGCG can counteract oxidative stress-induced changes of IVD cells* in vitro* via protection of the mitochondrial membrane from depolarization. In oxidative stress, EGCG also activated PI3K/Akt as an important prosurvival mechanism of IVD cells. As this study did not provide direct evidence whether and how the EGCG activated Akt and mitochondria protection are linked, investigation of this coupling in IVD cells will be an objective of future research.

Together with previously reported anti-inflammatory, anticatabolic, and analgesic effects of EGCG, our findings can be further used for the development of novel therapies targeting consequences of oxidative stress and neovascularization in degenerative disc disease.

## Supplementary Material

Supplementary figure 1. H_2_O_2_ sensitivity study, p21 expression during cell culture period, the effect of H_2_O_2_ on senescence-associated secretory phenotype and confirmation of the LY294002 activity/specificity.
Supplementary figure 2. The expression of p16 Ink4a in oxidative stress-induced senescence of IVD cells.
Supplementary figure 3. The role of reactive oxygen species and mitochondria in senescence and apoptosis of IVD cells (schematic).

## Figures and Tables

**Table 1:** Intervertebral disc pathologies used for primary cell cultures.

Donor information			Experiments	
Number	Gender, age	Level pathology, grade	Sublethal oxidative stress	Lethal oxidative stress
1	M, 35	L4/L5 Herniation IV	H_2_O_2_ sensitivity study, p21 time course Senescence validation 10 days: additive effect	H_2_O_2_ sensitivity study PI/A
2	F, 77	L4/L5 DDD III	p21 time course 10 days: additive effect	JC-1, MTT
3	F, 53	L4/L5 DDD IV	H_2_O_2_ sensitivity study, p21 time course Senescence validation 10 days: additive effect	H_2_O_2_ sensitivity study PI/A, MTT
4	F, 39	L5/S1 Herniation V	H_2_O_2_ sensitivity study Senescence validation 10 days: antioxidant	H_2_O_2_ sensitivity study PI/A, MTT
5	F, 31	L4/L5 Herniation uk	H_2_O_2_ sensitivity study Senescence validation	H_2_O_2_ sensitivity study JC-1, PI/A, MTT
6	F, 64	L4/L5 DDD III	H_2_O_2_ sensitivity study Senescence validation	H_2_O_2_ sensitivity study JC-1, PI/A, MTT
7	F, 53	L5/S1 Herniation IV	—	JC-1
8	M, 50	L4/5 DDD III	—	JC-1
9	F, 54	L5/S1 Herniation V	10 days: recovery, RT qPCR	—
10	M, 64	L5/S1 Herniation III	10 days: recovery, RT qPCR	—
11	M, 44	L5/S1 Her IV	10 days: antioxidant, RT qPCR	—
12	M, 56	L4/L5 Herniation III	10 days: recovery, RT qPCR	—
13	F, 51	C4/5 Herniation III	10 days: antioxidant	—
14	F, 42	L3/L4 Herniation III	10 days: antioxidant	—
15	F, 35	L5/S1 DDD, Herniation IV	10 days: recovery	—
16	M, 41	L4/L5 DDD III	10 days: additive effect	—
17	M, 40	L5/S2 DDD, Herniation V	10 days: antioxidant	—
18	M, 50	L4/L5 DDD, Herniation III	10 days: additive effect	—
19	F, 42	L3/L4 Herniation III	10 days: recovery	—
20	M, 43	L5/S1 DDD, uk	—	JC-1, bright field, MTT, MTT (I)
21	F, 51	L4/L5 DDD IV	—	JC-1, bright field, MTT, MTT (LY), MTT (I)
22	M, 47	L4/L5 DDD, Herniation III	—	JC-1, bright field, MTT
23	F, 44	L4/L5 DDD III	—	JC-1, bright field, MTT, MTT (LY), MTT (I)
24	F, 43	L5/S1 DDD, Herniation III	—	JC-1, bright field, MTT, MTT (LY), MTT (I)
25	M, 47	L4/5 DDD, Herniation III	—	Lethal: MTT (LY), MTT (I)
26	M, 39	L4/5 DDD, Herniation IV	—	Lethal: MTT (LY), MTT (I)
27	M, 21	L5/S1 Herniation III	—	Lethal: MTT (LY), MTT (I)
28	F, 43	L4/5 DDD, Herniation IV	—	Lethal: MTT (LY), MTT (I)
29	M, 33	L5/S1 Herniation III	—	Lethal: WB
30	F, 39	L5/S1 DDD, Herniation III	—	Lethal: MTT (LY), WB, MTT (I)
31	F, 39	L4/5 DDD, Herniation IV	—	Lethal: MTT (LY), WB, MTT (I)
32	uk	uk	—	Lethal: MTT (LY), WB
33	M, 55	L3/4 DDD III	—	Lethal: WB
34	F, 56	L3/4 Herniation IV	—	Lethal: MTT (LY), WB
35	M, uk	L4/5 Herniation uk	—	Lethal: MTT (LY)
36	uk	uk	—	Lethal: MTT (LY)

DDD: degenerative disc disease, PI/A: Propidium Iodide/Annexin staining, MTT: MTT assay, MTT (LY): MTT assay with LY, MTT (I): MTT assay with insulin, and uk: unknown.

**Table 2:** Experimental design.

Sublethal oxidative stress, induction of premature senescence			
Experimental setup	Stress phase (2 hours)	Recovery phase (up to 15 days)	Tested EGCG effect
(1) Antioxidant	50 *μ*M H_2_O_2_ + 10 *μ*M EGCG	—	ROS neutralization
(2) Recovery	50 *μ*M H_2_O_2_	+10 *μ*M EGCG	Interaction with poststress signaling (e.g., changes in gene expression)
(3) Combined	50 *μ*M H_2_O_2_ + 5 *μ*M EGCG	+5 *μ*M EGCG	ROS neutralization Interaction with poststress signaling

Lethal oxidative stress, cell death induction			
Experimental setup	Stress phase (24 hours)		Tested EGCG effect

(1) Survival	100 and 200 *μ*M H_2_O_2_ + 10 *μ*M EGCG		Prosurvival Antioxidant

## Abbreviations

A: Annexin V-FITC
AF: Annulus fibrosus
Akt: Protein kinase B
EGCG: Epigallocatechin 3-gallate
IL: Interleukin
IVD: Intervertebral disc
JC-1: 5,5′,6,6′-Tetrachloro-1,1′,3,3′-tetraethylbenzimidazolcarbocyanine iodide
LY: LY294002
MMP: Matrix metalloproteinase
MTT: 3-[4,5-Dimethylthiazol-2-yl]-2,5-diphenyl tetrazolium bromide
NF-*κ*B: Nuclear factor kappa-light-chain-enhancer of activated B cells
NP: Nucleus pulposus
PI: Propidium Iodide
ROS: Reactive oxygen species
SA *β*-gal: Senescence-associated *β*-galactosidase
SASP: Senescence-activated secretory phenotype.
