# Supplementary material for: The Natural Polyphenol Epigallocatechin Gallate Protects Intervertebral Disc Cells from Oxidative Stress
Source: Oxid Med Cell Longev. 2016 Mar 28;2016:7031397. doi: 10.1155/2016/7031397 (PMC4826942; doi:10.1155/2016/7031397)

Additional file 1. H_2O_2 sensitivity study, p21 expression during cell culture period, the effect of H_2O_2 on senescence-associated secretory phenotype and confirmation of the LY294002 activity/specificity

IVD cells were treated with different concentrations of H_2O_2 to determine the cellular metabolic activity by MTT assay. **(A)** After 24 hours 50, 100 and 200 µM H_2O_2 caused a significant decrease in the metabolic activity. Thus concentrations 100 and 200 µM were selected for lethal oxidative stress induction (n = 5). **(B)** Metabolic activity of IVD cells treated with 50 µM H_2O_2 for 2 hours did not change within 3 days, therefore this concentration was chosen for sub-lethal oxidative stress induction (n = 5). **(C)** The expression of p21 on day 1, 5, 10 and 15 was tested by immunoblotting, with etoposide (10 µM, 72 hours) as a positive control. p21 expression increased during the cell culture period as an artifact of the *in vitro* environment (n = 3). **(D)** Sub-lethal oxidative stress (50 µM H_2O_2, 2 hours) did not increase the expression of inflammatory markers (IL-6, IL-8) and catabolic enzymes (MMP1, MMP3, MMP13) on day 1 post-stress (n = 4). **(E)** Akt activator insulin at 0.5 µl/mL increased proliferation of IVD cells whereas addition of 10 µM LY294002 (LY) abolished this effect, confirming functionality of LY for further tests involving Akt. Asterisks indicate statistical significance p < 0.05 (ANOVA, Tukey post-hoc).

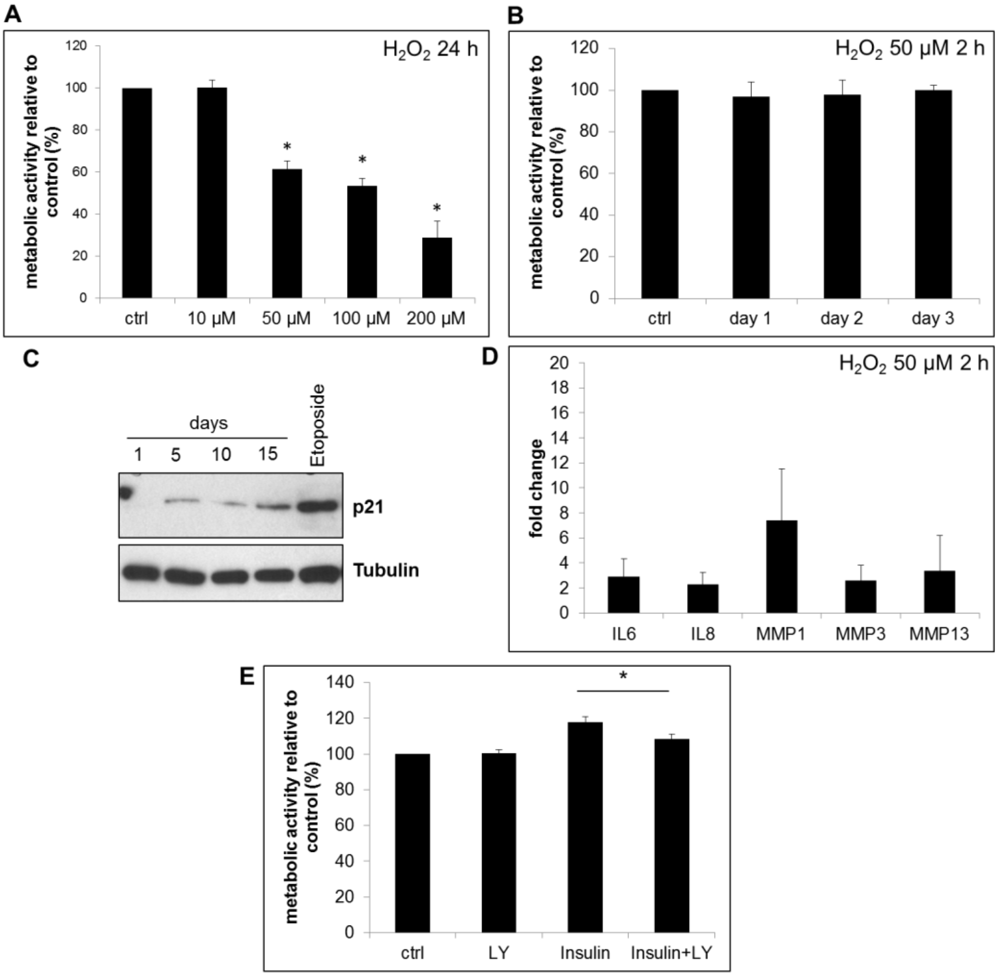

Additional file 2. The expression of p16 Ink4a in oxidative stress-induced senescence of IVD cells

Senescence was induced with 50 µM H_2O_2 for 2 hours and 10 µM EGCG was added directly to the stress phase (antioxidant experimental setup). The expression of p16 was measured on day 10 post-stress with SAOS-2 cells as positive control. **(A)** The expression of p16 is constitutively active in SAOS-2 cells. The expression of p16 was not induced by H_2O_2 in 3 out of 4 donors, therefore the effects of EGCG could not be studied (n = 4). **(B)** The constitutive expression of p16 in SAOS-2 cells is not affected by 10 µM EGCG.

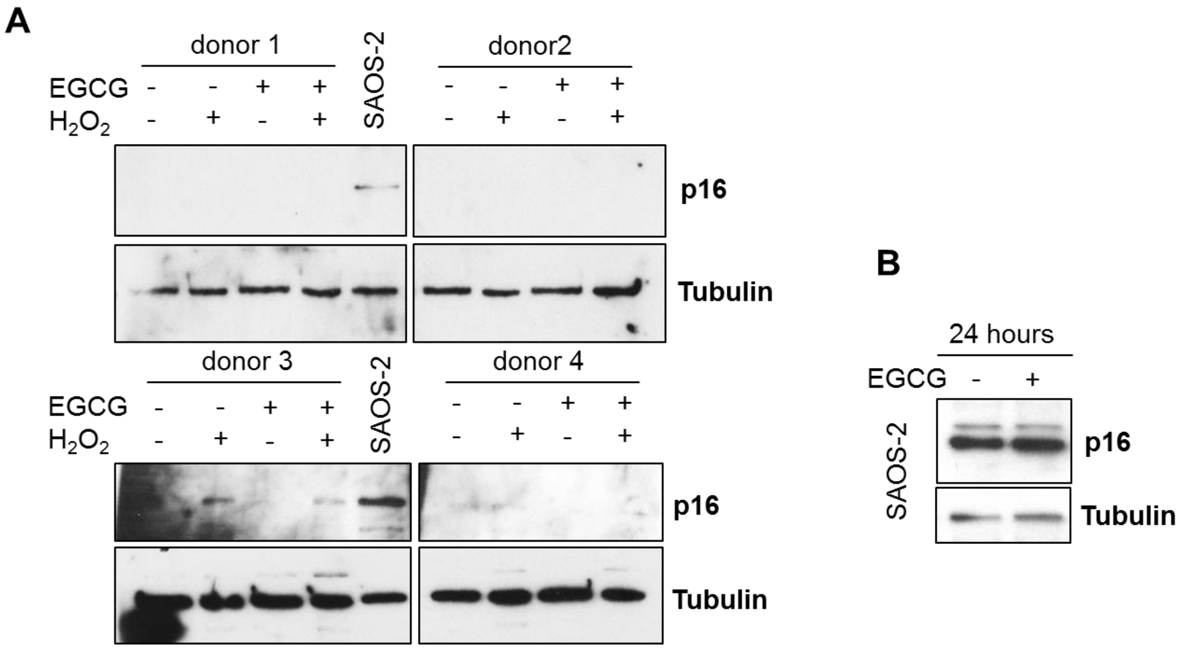

Additional file 3. The role of reactive oxygen species and mitochondria in senescence and apoptosis of IVD cells (schematic)

(A, C) Sub-lethal oxidative stress causes damage of cellular macromolecules, which activates the p53-p21 pathway and growth arrest, without significant mitochondrial depolarization. During growth arrest, cells can repair the damage or irreversibly enter senescence, depending on various factors, such as activity of cellular antioxidants and inflammatory pathways. **(B, D)** Lethal oxidative stress leads to extensive mitochondrial membrane depolarization and subsequent mitochondrial ROS leakage, which induces apoptosis. Based on our data, EGCG can protect mitochondrial membrane from ROS leakage and prevent subsequent activation of cell death cascade. **(C, D)** The ratio of red/green fluorescence, with a loss of mitochondrial membrane potential in the H_2O_2 treatment groups is shown. Data presented in (D) are replicated from Figure 5D to emphasize the difference between the mitochondrial membrane potential in senescence and apoptosis. Asterisks indicate statistical significance at $p < 0.05$ (ANOVA, Tukey post-hoc) (n = 5).

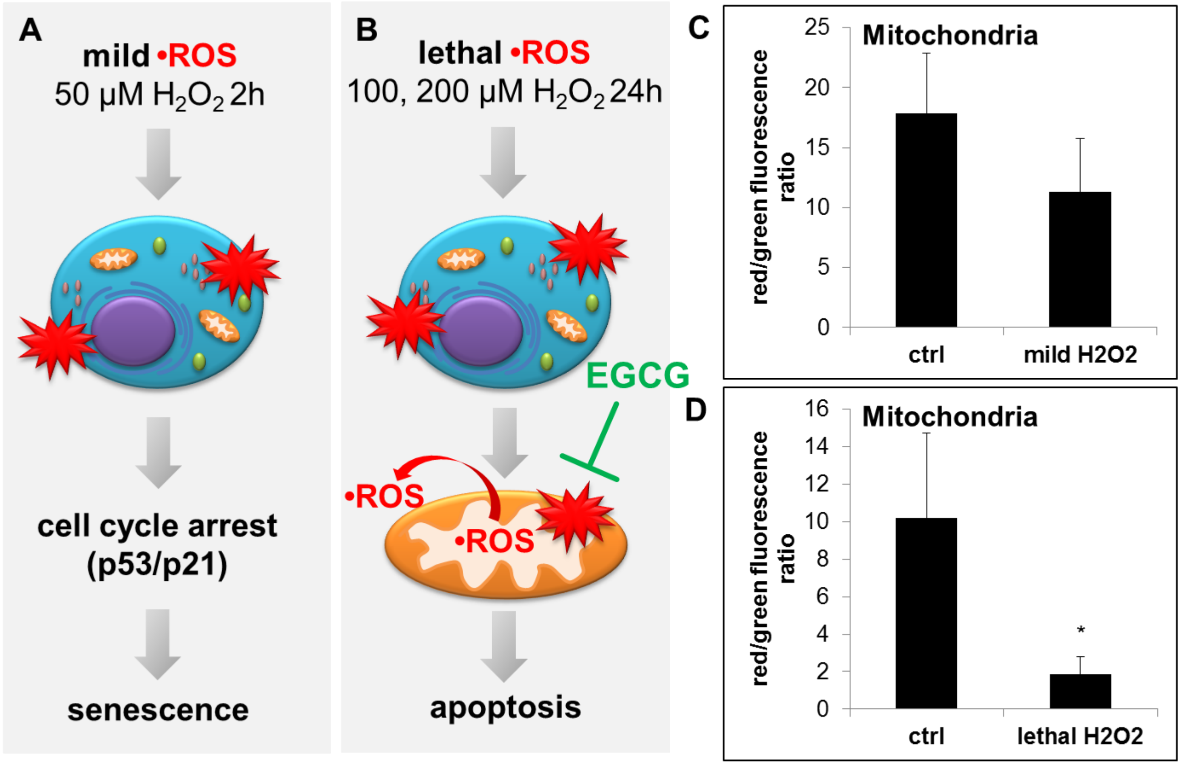

Supplement: Supplementary file 1 — Supplementary figure 1. H2O2 sensitivity study, p21 expression during cell culture period, the effect of H2O2 on senescence-associated secretory phenotype and confirmation of the LY294002 activity/specificity. Supplementary figure 2. The expression of p16 Ink4a in oxidative stress-induced senescence of IVD cells. Supplementary figure 3. The role of reactive oxygen species and mitochondria in senescence and apoptosis of IVD cells (schematic). [file 7031397.f1.pdf]
